# The Verification Gap: Artificial Intelligence Adoption, Hallucination Awareness, and Verification Practices Among Medical Trainees and Professionals in Pakistan

**DOI:** 10.7759/cureus.117238

**Published:** 2026-09-30

**Authors:** Mobeen Sajjad, Sammeullah Mirza, Ahsan Sajjad, Rida Amer

**Affiliations:** 1 Department of Medicine, Saad Hospital and Cardiac Care Center, Daska, PAK; 2 Department of Medicine, Ibn Sina Wilcrest Community Medical Clinic, Houston, USA; 3 Department of Internal Medicine, Prolato Clinical Research Center, Houston, USA

**Keywords:** ai literacy, artificial intelligence, hallucination, large language models, lmic, medical research, pakistan, research integrity, verification

## Abstract

Background

Artificial intelligence (AI) tools have been rapidly adopted by medical researchers, yet whether medical trainees and professionals in low- and middle-income countries possess the awareness and habits needed to use these tools safely remains poorly documented. This study characterized AI adoption, hallucination awareness, and verification and disclosure practices among medical trainees and professionals in Pakistan.

Methods

A cross-sectional anonymous online survey was conducted among medical students, house officers, residents, physicians, and faculty involved in research or academic work across Pakistan (May 2026). Descriptive statistics and chi-square tests were applied to 373 eligible responses.

Results

AI use was near-universal (99.7%), with 60.3% using AI daily. The most commonly reported tool was Claude (Anthropic, San Francisco, CA) (40.5%), followed by ChatGPT (OpenAI, San Francisco, CA) (29.2%), and Perplexity (Perplexity AI, Inc., San Francisco, CA) (26.0%), though this ranking likely reflects sampling characteristics. Despite high adoption, 59.2% (95% CI: 54.2-64.1%) typically did not verify AI outputs before use, and 40.2% had never heard that AI can generate fabricated references. In behavioral vignettes, 36.5% assumed convincing AI-generated references were authentic, and 54.2% would continue using remaining AI content after discovering one fabricated reference. Formal research training was strongly associated with consistent disclosure (51.7% vs. 17.1%; χ²(1) = 48.43, p < 0.001, Cramer's V = 0.36). Role was significantly associated with both verification behavior (χ²(4) = 17.22, p = 0.0018, Cramer’s V = 0.22) and hallucination awareness (χ²(4) = 13.44, p = 0.0093, Cramer’s V = 0.19), with non-verification highest among medical students and unawareness highest among physicians. Daily use frequency, research training, and publication count showed no other significant associations.

Conclusions

Medical trainees and professionals in Pakistan demonstrate high AI adoption alongside incomplete hallucination awareness and infrequent verification - a pattern that may carry implications for research integrity. Formal training was the only factor significantly associated with consistent disclosure. Integration of AI literacy into medical curricula and institutional governance frameworks merits consideration.

## Introduction

The integration of artificial intelligence (AI) tools, particularly large language models (LLMs) such as ChatGPT (OpenAI, San Francisco, CA), Claude (Anthropic, San Francisco, CA), and Perplexity (Perplexity AI, Inc., San Francisco, CA), into medical research workflows has accelerated considerably in recent years [1,2]. These tools offer practical advantages for researchers working under resource constraints. They can assist with literature synthesis, manuscript drafting, language editing, data interpretation, and reference generation at a speed and scale previously unavailable to independent investigators in low- and middle-income country (LMIC) settings [3,4].

However, a recognized limitation of contemporary LLMs is their tendency to generate content that appears scientifically credible but is factually incorrect - a phenomenon commonly referred to as hallucination [5,6]. AI hallucination refers to the confident generation of false, fabricated, or unsupported information by an LLM, presented with the same fluency and apparent authority as accurate content. Of particular relevance to research integrity is the generation of fabricated references: citations that appear authentic in format, authorship, and journal attribution, but do not correspond to real publications [7]. When incorporated into submitted manuscripts without verification, such fabricated references may compromise the reliability of the scientific record [8]. Hallucinations have been categorized into three primary types: input-conflicting hallucination, where outputs fail to align with the user's prompt; context-conflicting hallucination, where outputs contradict earlier content within the same response; and fact-conflicting hallucination, where outputs contradict established, verifiable facts [9]. Fabricated references, the primary concern of this study, represent a specific manifestation of fact-conflicting hallucination.

Existing literature on AI use in medical research has predominantly focused on adoption rates, perceived utility, and ethical attitudes [1,2,10]. Systematic documentation of verification practices and hallucination awareness remains limited, particularly in LMIC contexts [10]. Pakistan presents an important setting: it maintains one of the largest medical education systems in South Asia, has experienced notable growth in research output, and yet lacks formal AI governance frameworks or structured AI literacy training in most medical institutions [11].

This study was designed to address this gap. The primary objective of this study was to describe the prevalence of AI adoption, hallucination awareness, and verification and disclosure practices among medical trainees and professionals in Pakistan, encompassing medical students, house officers, residents, physicians, and faculty engaged in medical education, clinical practice, research, or academic work. As a secondary, exploratory objective, we examined associations between these practices and demographic and behavioral factors, including professional role, AI use frequency, formal research training, and publication history. We hypothesized that AI adoption would be high while verification and disclosure practices would remain comparatively underdeveloped, a discrepancy we term the verification gap, and that these practices would vary by professional role and other factors examined. Given the current absence of structured AI literacy training and governance frameworks in Pakistani medical education, these findings may hold practical value for informing future curriculum development and institutional policy discussions.

This article was previously posted to the medRxiv preprint server on May 30, 2026 [12].

## Materials and methods

Study design

We conducted a cross-sectional, self-administered, anonymous online survey, reported in accordance with the Strengthening the Reporting of Observational Studies in Epidemiology (STROBE) guidelines [13]. We use the term “verification gap” throughout this manuscript to refer to the discrepancy between the level of AI adoption observed and the level of independent verification, hallucination awareness, or disclosure practice observed within the same cross-sectional sample; this term describes a coexisting discrepancy at a single time point and does not imply a measured change in either variable over time.

Setting and participants

Participants were recruited from across Pakistan via WhatsApp (Meta Platforms, Inc., Menlo Park, CA), Instagram (Meta Platforms, Inc.), Facebook (Meta Platforms, Inc.), and LinkedIn (Microsoft Corporation, Redmond, WA) networks during May 2026. Eligible participants were individuals currently involved in medical education, clinical practice, research, or academic work in Pakistan, aged 18 years or older, who provided voluntary informed consent.

Survey instrument

The survey was administered using Google Forms (Google, Mountain View, CA) and comprised 24 items across four domains: (1) demographic and professional characteristics; (2) AI adoption patterns; (3) verification practices and hallucination awareness, including two behavioral vignettes presenting realistic AI use scenarios; and (4) attitudes and disclosure practices. Verification practice was defined as the participant's self-reported approach toward independently checking AI-generated content, including references, before incorporating it into academic or research work. Hallucination awareness was defined as the participant's prior familiarity with the phenomenon of AI models generating fabricated or unsupported scientific content, including non-existent references. Disclosure practice was defined as the participant's self-reported tendency to disclose AI assistance when preparing manuscripts or research documents, as required by many journal policies. Questionnaire items were developed by the investigator based on a review of published literature on AI adoption in medical research and clinical practice, combined with the investigator's own experience with AI tools in academic work. The draft instrument was reviewed by two co-investigators (Dr. Sammeullah Baig Mirza and Ahsan Sajjad, MD) prior to deployment. Rather than a separate pre-launch pilot, the instrument underwent iterative refinement during initial live deployment: after the first 24 responses were collected, three items (gender, purpose of AI use, and disclosure practice) were identified as missing and added to the instrument. These initial respondents were individually recontacted to provide responses to the added items, which were then incorporated into the final dataset. As all three items were retrospectively incorporated for the initial 24 respondents prior to analysis, no missing data resulted from their later addition. However, as disclosure practice was among this study's primary outcomes, its introduction only after initial piloting represents a limitation of iterative instrument design that should be considered when interpreting this finding. To reduce potential sources of bias, the survey was administered anonymously in a standardized online format, without collection of personal identifiers; participation was voluntary, which together were intended to reduce social desirability bias in self-reported verification and disclosure practices. The full survey instrument, including exact item wording and response options, is provided in the Appendices.

Ethical considerations

This study involved anonymous, voluntary participation with no collection of personal identifiers, no clinical intervention, and no patient data. All participants provided explicit informed consent. The study was reviewed by the Saad Hospital & Cardiac Care Center review committee and determined exempt from full ethical review, consistent with standard criteria for anonymous, minimal-risk survey research. The author declares that AI assistance was used in preparing this manuscript to assist with drafting and language refinement; all study design, data analysis, interpretation, and factual content were independently verified by the author, including manual verification of every cited reference.

Statistical analysis

Data were analyzed using Python (pandas and scipy libraries) (Python Software Foundation, Wilmington, DE). Frequencies and percentages were calculated for all categorical variables. Chi-square tests of independence assessed associations between demographic and training variables and key outcomes, including verification behavior, hallucination awareness, and disclosure practice. Effect sizes for chi-square tests were quantified using Cramer's V and interpreted according to Cohen's conventional benchmarks (~0.1 small, ~0.3 medium, ~0.5 large). A p-value of less than 0.05 was considered statistically significant.

For chi-square analyses, categorical outcomes were dichotomized as follows: “non-verification” comprised respondents selecting “I use AI mainly to save time, energy, and effort and usually do not independently verify outputs before use,” with all other response options for this item (including partial or comprehensive verification, and trust in convincing outputs) coded as the comparison category. “Unawareness” comprised respondents selecting “I regularly use AI but had never heard of the concept that AI may generate fabricated scientific information,” with all other response options coded as aware. “Always discloses” comprised respondents selecting “Yes, always,” with “Sometimes,” “Never,” and other response options coded as the comparison category. Daily AI use frequency was dichotomized as “Daily” versus all other frequencies combined (“Weekly,” “Monthly,” “Rarely,” “Never”). Publication count was dichotomized as three or more publications versus fewer than three.

Missing responses were handled using complete-case analysis for each individual variable; respondents with missing data on a given item were excluded only from analyses involving that specific item. For chi-square tests involving professional role, the single respondent in the “Other” category was excluded due to an expected cell count below the conventional threshold of five; this respondent remains included in all descriptive tables and frequencies.

As this exploratory survey used open online recruitment and no prior estimate of the primary outcome was available, an a priori sample size calculation was not performed. Recruitment remained open throughout the predefined study period (May 2026), yielding 373 eligible responses. Given the number of comparisons conducted across demographic and behavioral variables, adjustment for multiple comparisons was not performed, as this study was exploratory and hypothesis-generating in nature rather than confirmatory. All statistical findings, including those reaching conventional significance thresholds, should therefore be interpreted cautiously and considered hypothesis-generating pending confirmation in future studies.

## Results

Participant characteristics

A total of 373 individuals completed the survey, all confirming active involvement in medical education, clinical practice, research, or academic work in Pakistan. No responses were excluded; minor item-level non-response (one to three missing values per variable) accounts for denominators slightly below 373 in some rows of Table 1. Participant characteristics are summarized in Table 1, and the participant flow through recruitment and instrument revision is presented in Figure 1.

**Table 1:** Demographic and professional characteristics of the study participants (N = 373). Values are presented as n (%). Percentages are based on the total study population (N = 373). Minor item-level missing responses resulted in denominators slightly below 373 for selected variables, as described in the Results. This table presents descriptive frequencies only; no statistical comparisons were performed on these data.

Characteristic	N	%
Current role: Medical student	99	26.5
Current role: Resident	106	28.4
Current role: House officer	62	16.6
Current role: Physician	63	16.9
Current role: Faculty	42	11.3
Current role: Other	1	0.3
Gender: Female	186	49.9
Gender: Male	185	49.6
Publications: 0	1	0.3
Publications: 1–2	240	64.3
Publications: 3–5	93	24.9
Publications: >5	38	10.2
Formal research training: Yes	151	40.5
Formal research training: No	219	58.7
Formal research training: Unsure	3	0.8
Previously participated in research: Yes	362	97.0
Previously participated in research: No	11	2.9

**Figure 1:**
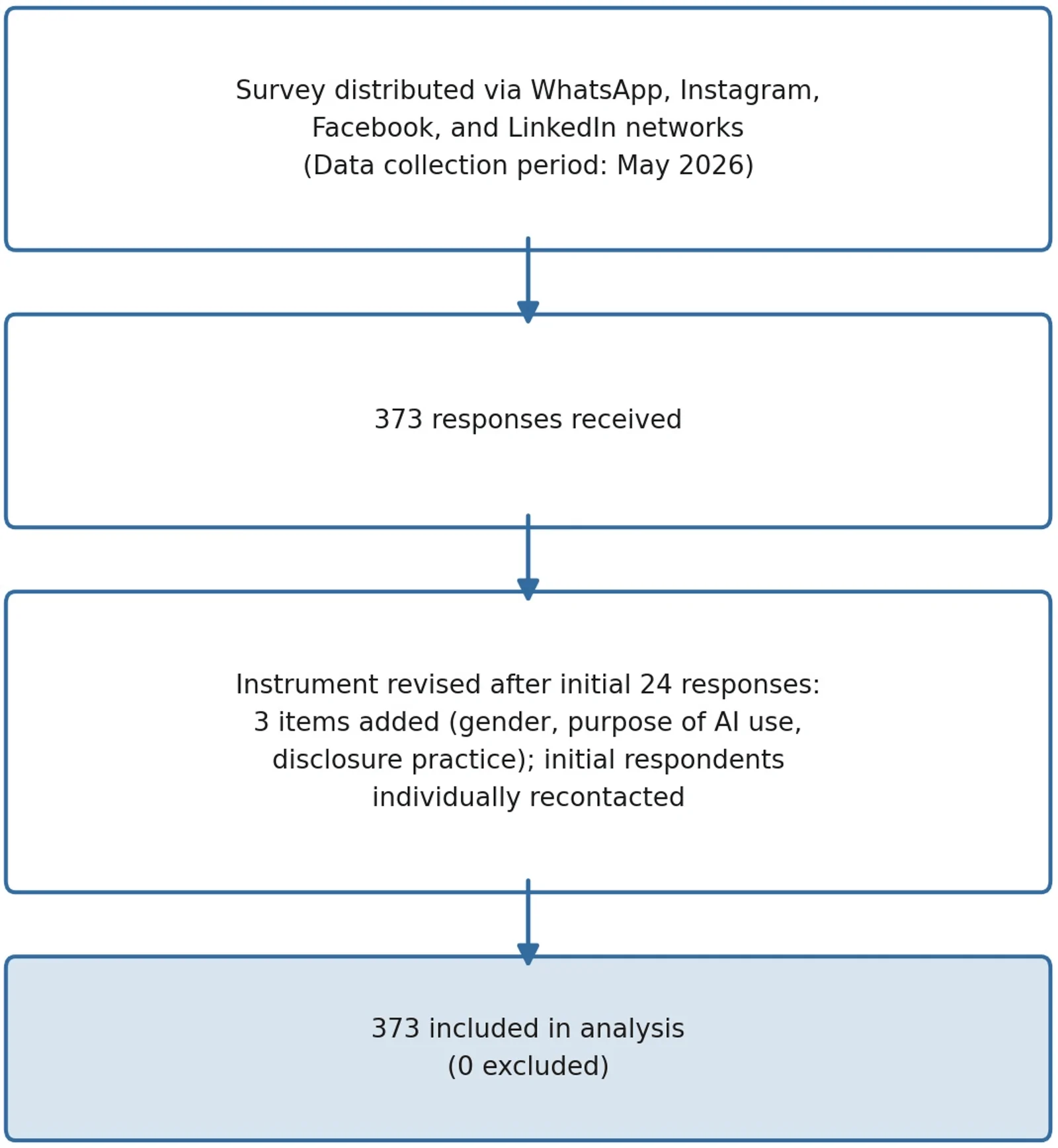
Participant flow through recruitment, instrument revision, and inclusion in analysis. All 373 respondents who completed the survey were included in the final analysis; no responses were excluded. Three items (gender, purpose of AI use, disclosure practice) were added to the instrument after the first 24 responses were collected, and these initial respondents were individually recontacted to complete the added items.

The most common roles were resident (28.4%, n = 106) and medical student (26.5%, n = 99), followed by physician (16.9%, n = 63), house officer (16.6%, n = 62), faculty (11.3%, n = 42), and other (0.3%, n = 1). Gender distribution was approximately equal (female = 49.9%, male = 49.6%). Responses were received from 11 cities, with the largest proportions from Gujranwala (19.6%), Lahore (17.7%), and Faisalabad (13.4%). The majority (64.3%, n = 240) reported one to two publications; 24.9% reported three to five, and 10.2% reported more than five publications. Formal research methodology training had been received by 40.5% (n = 151), 58.7% (n = 219) reported no such training, and 0.8% (n = 3) were unsure. The large majority (97.0%, n = 362) reported having previously participated in research.

AI adoption patterns

AI use was near-universal: 99.7% reported using at least one AI tool. Daily use was reported by 60.3% (n = 225), weekly by 25.5% (n = 95), and monthly by 12.1% (n = 45). The most commonly reported tool in this sample was Claude (40.5%, n = 151), followed by ChatGPT (29.2%, n = 109) and Perplexity (26.0%, n = 97). This ranking likely reflects characteristics of the recruitment network and should not be interpreted as reflecting national AI adoption patterns. Common purposes for AI use included literature search, generating or finding references, interpreting data, and writing and editing manuscripts.

Verification practices

Despite near-universal adoption, independent verification of AI outputs before use was uncommon. The majority (59.2%, n = 221) reported typically not verifying outputs; a further 31.6% (n = 118) verified only selected outputs or references rather than verifying comprehensively or not at all. Only 1.3% (n = 5) verified nearly all AI-generated content before use, and 7.2% (n = 27) reported trusting most AI outputs if they appeared scientifically convincing; the distribution of verification approaches is presented in Figure 2.

**Figure 2:**
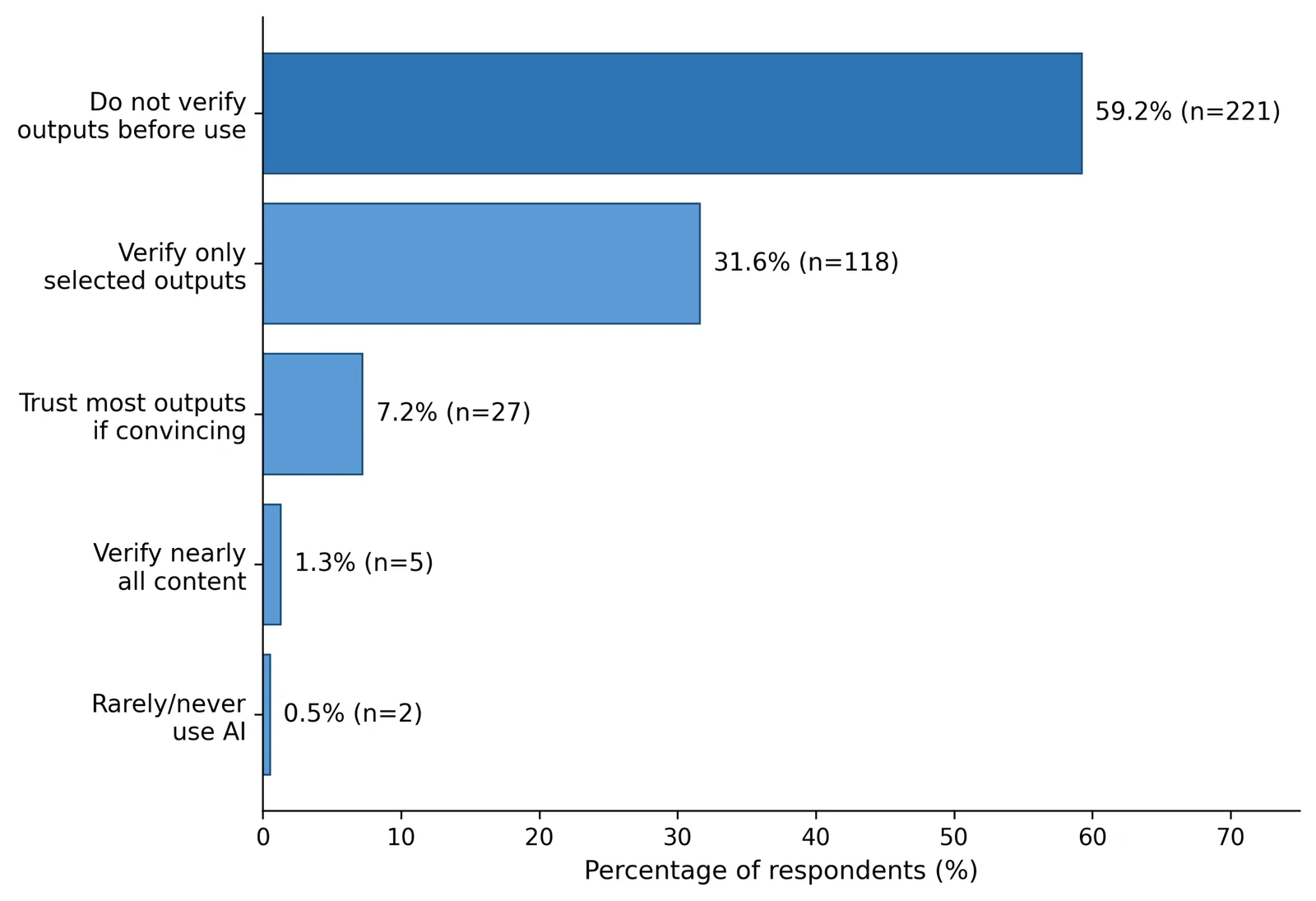
Verification practices among survey respondents (N = 373). Bars show the percentage of respondents reporting each verification approach toward AI-generated content before incorporating it into academic or research work, ranked from most to least common.

In a behavioral vignette presenting AI-generated text with convincing-appearing references, 36.5% (n = 136) assumed authenticity based on appearance alone, while 35.1% (n = 131) recognized the need for independent verification; a further 27.6% (n = 103) considered the references probably correct but felt important ones should be checked. In a second vignette, 54.2% (n = 202) would remove only the discovered fabricated citation and continue using remaining AI output, 26.5% (n = 99) would verify all remaining AI-generated content before further use, and 16.9% (n = 63) would continue because occasional errors are expected. When asked about the speed-versus-reliability tradeoff, 49.6% (n = 185) would prioritize speed even if some risk remained; 21.2% (n = 79) would prioritize reliability even at the cost of efficiency.

Chi-square analysis revealed a statistically significant association between professional role and verification behavior (χ²(4) = 17.22, p = 0.0018, Cramer’s V = 0.22). The single respondent in the “Other” role category was excluded from this analysis due to an expected cell count below five; this respondent is retained in all descriptive tables. Non-verification was highest among medical students (73.7%, 95% CI: 64.3-81.4%) and lowest among residents (46.2%, 95% CI: 37.0-55.7%), with house officers (64.5%), faculty (57.1%), and physicians (55.6%) falling between these extremes. No statistically significant association was found between verification behavior and daily versus non-daily use frequency (χ²(1) = 0.22, p = 0.64), formal research training (χ²(1) = 1.02, p = 0.31), or number of publications (χ²(1) = 0.17, p = 0.68).

Hallucination awareness

Awareness of AI hallucination was substantially incomplete. Among respondents, 40.2% (n = 150) reported regularly using AI but having never heard that AI may generate fabricated scientific references; 27.9% (n = 104) knew AI could make mistakes but had not considered fabricated references specifically; and 31.1% (n = 116) reported prior full awareness; the distribution of awareness levels is presented in Figure 3.

**Figure 3:**
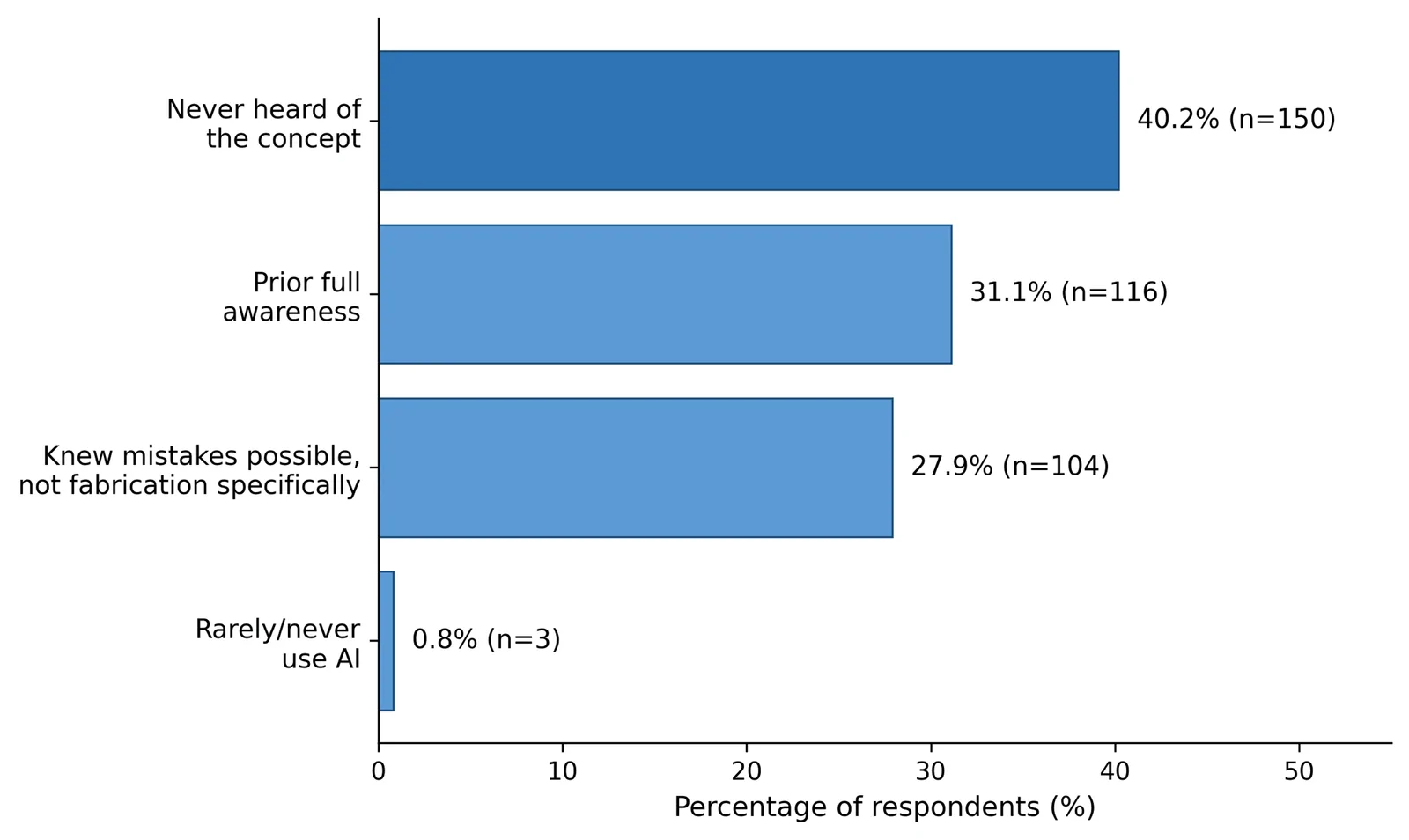
Hallucination awareness among survey respondents (N = 373). Bars show the percentage of respondents reporting each level of prior awareness that AI models can generate fabricated or unsupported scientific content, ranked from most to least common.

A statistically significant association was found between professional role and awareness (χ²(4) = 13.44, p = 0.0093, Cramer’s V = 0.19), again excluding the single “Other” respondent for the same reason: unawareness was highest among physicians (50.8%, 95% CI: 38.8-62.7%) and house officers (51.6%), intermediate among medical students (41.4%), and faculty (33.3%), and lowest among residents (28.3%, 95% CI: 20.6-37.5%). No significant association was found between daily AI use and awareness (χ²(1) = 0.74, p = 0.39). A comparison of non-verification and unawareness rates across professional roles is presented in Figure 4.

**Figure 4:**
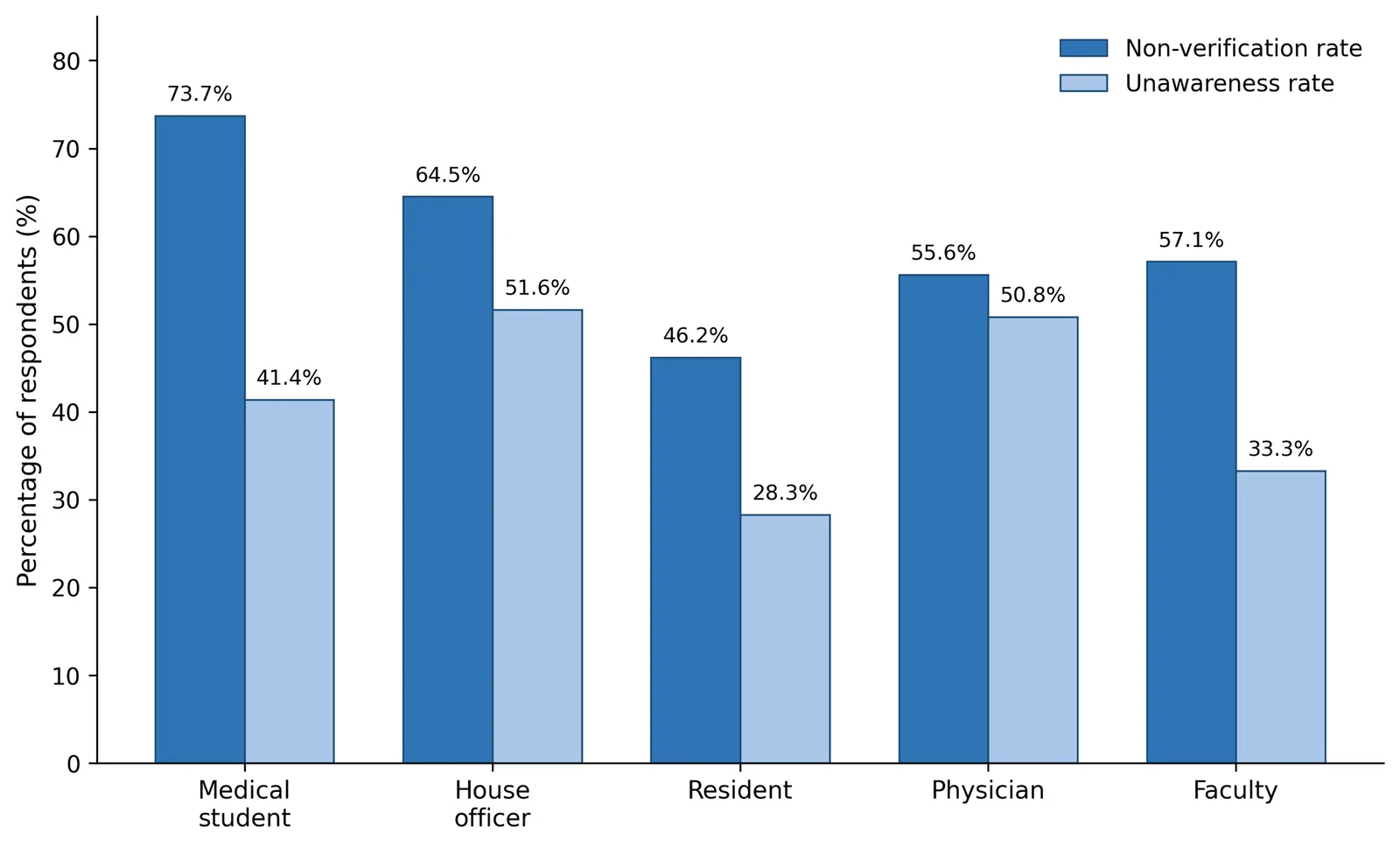
Non-verification and unawareness rates by professional role (N = 373). Bars show the percentage of respondents in each professional role who reported not verifying AI-generated content before use (non-verification rate) and who reported no prior awareness that AI can generate fabricated content (unawareness rate). Both associations were statistically significant (verification: χ²(4) = 17.22, p = 0.0018; awareness: χ²(4) = 13.44, p = 0.0093).

Despite limited conceptual awareness, 85.8% (n = 320) reported personally encountering AI-generated information that proved incorrect or fabricated after verification, with this experience changing how they used AI. A further 8.0% (n = 30) reported the same encounter but continued using AI in the same way. The remaining 6.2% (n = 23) reported either suspecting but never confirming such an encounter (n = 15), no encounter at all (n = 4), or regular AI use without ever having actively checked for this possibility (n = 4).

Attitudes and disclosure practices

Over half of respondents (55.5%, n = 207) viewed AI-generated content as generally reliable when it appeared convincing and included references; a further 27.1% (n = 101) viewed AI as useful but felt content should usually be independently verified, and 16.1% (n = 60) felt AI outputs should never be trusted without verification. Regarding disclosure, 22.3% (n = 83) reported never disclosing AI assistance in manuscripts, and 1.3% (n = 5) were unaware that disclosure is required. Only 31.1% (n = 116) reported always disclosing; 44.8% (n = 167) disclosed only sometimes.

A statistically significant association was observed between formal research methodology training and consistent AI disclosure: 51.7% (95% CI: 43.7-59.5%) of trained researchers reported always disclosing, compared with 17.1% (95% CI: 12.5-22.4%) of untrained researchers (χ²(1) = 48.43, p < 0.001, Cramer's V = 0.36). This was the strongest and most robust association identified in this dataset.

Survey-related attitude change

When asked whether completing the survey changed their perspective on verifying AI-generated content, 46.4% (n = 173) reported being more likely to verify outputs following participation, while a comparably large proportion, 35.1% (n = 131), reported no change in perspective; 17.4% (n = 65) reported already routinely verifying, and 1.1% (n = 4) were unsure. A summary of these key findings is presented in Table 2. Associations between demographic and behavioral variables and the study’s key outcomes, including the underlying dataset for these comparisons, are presented in Table 3. Corresponding chi-square test statistics and effect sizes for each comparison are presented in Table 4.

**Table 2:** Key findings: AI adoption, verification, hallucination awareness, and disclosure (n = 373). Values are presented as numbers (N) and percentages (%). Claude was the most frequently reported AI tool in this sample; this ranking likely reflects characteristics of the recruitment network and should not be interpreted as representative of national AI adoption patterns. This table presents descriptive frequencies only; no statistical comparisons were performed on these data. Associations between these variables and demographic or behavioral factors are tested separately in Table 3.

Finding	N	%
Use AI daily	225	60.3
Never use AI	1	0.3
Most reported tool: Claude	151	40.5
Most reported tool: ChatGPT	109	29.2
Most reported tool: Perplexity	97	26.0
Do not verify AI outputs before use	221	59.2
Verify only selected outputs or references	118	31.6
Verify nearly all AI content	5	1.3
Assume convincing AI references are authentic (Vignette 1)	136	36.5
Consider references probably correct, check important ones (Vignette 1)	103	27.6
Would remove only incorrect reference and continue (Vignette 2)	202	54.2
Would verify all remaining AI content (Vignette 2)	99	26.5
Never heard AI can fabricate references	150	40.2
Encountered incorrect AI content (changed behavior)	320	85.8
View AI content as reliable if convincing	207	55.5
View AI as useful but usually needing verification	101	27.1
Never disclose AI use in manuscripts	83	22.3
Unaware disclosure is required	5	1.3
More likely to verify after completing survey	173	46.4
No change in perspective after completing survey	131	35.1

**Table 3:** Dataset for associations between demographic and behavioral variables and key outcomes (N = 373). Values are presented as n/N and percentage (%) for each category. Corresponding chi-square test statistics and effect sizes for these comparisons are presented in Table 4. The single respondent in the “Other” professional role category (Table 1) is excluded from the professional role rows above due to an insufficient expected cell count for inferential analysis, as described in the Statistical analysis section.

Variable	Category	Outcome	n/N	%
Professional role	Medical student	Non-verification	73/99	73.7
Professional role	Resident	Non-verification	49/106	46.2
Professional role	House officer	Non-verification	40/62	64.5
Professional role	Physician	Non-verification	35/63	55.6
Professional role	Faculty	Non-verification	24/42	57.1
Professional role	Medical student	Unawareness	41/99	41.4
Professional role	Resident	Unawareness	30/106	28.3
Professional role	House officer	Unawareness	32/62	51.6
Professional role	Physician	Unawareness	32/63	50.8
Professional role	Faculty	Unawareness	14/42	33.3
Daily AI use frequency	Daily	Non-verification	136/225	60.4
Daily AI use frequency	Non-daily	Non-verification	85/148	57.4
Daily AI use frequency	Daily	Unawareness	86/225	38.2
Daily AI use frequency	Non-daily	Unawareness	64/148	43.2
Formal research training	Yes	Non-verification	85/151	56.3
Formal research training	No	Non-verification	136/219	62.1
Formal research training	Yes	Always discloses	78/151	51.7
Formal research training	No	Always discloses	37/219	16.9
Publications (3 or more vs. less than 3)	3 or more	Non-verification	80/131	61.1
Publications (3 or more vs. less than 3)	Less than 3	Non-verification	141/242	58.3

**Table 4:** Chi-square test statistics and effect sizes for associations presented in Table 3 (N = 373). Cramer’s V is the effect size for each chi-square test, interpreted per Cohen’s conventional benchmarks (approximately 0.1 small, 0.3 medium, 0.5 large). Each row reports the test statistic and effect size once for the comparison described in the corresponding rows of Table 3. df = degrees of freedom.

Variable	Outcome	Chi-square (χ²)	df	P-value	Cramer’s V
Professional role	Non-verification	17.22	4	0.0018	0.22
Professional role	Unawareness	13.44	4	0.0093	0.19
Daily AI use frequency	Non-verification	0.22	1	0.64	0.02
Daily AI use frequency	Unawareness	0.74	1	0.39	0.05
Formal research training	Non-verification	1.02	1	0.31	0.05
Formal research training	Always discloses	48.43	1	<0.001	0.36
Publications (3 or more vs. less than 3)	Non-verification	0.17	1	0.68	0.02

## Discussion

This cross-sectional survey of 373 medical trainees and professionals across Pakistan suggests a meaningful discrepancy between the intensity of AI adoption and the verification and disclosure practices needed to support its responsible use in academic research. AI use was near-universal and daily, yet hallucination awareness was substantially incomplete, independent verification was infrequent, and AI use was regularly undisclosed in manuscripts. These patterns varied significantly by professional role for both verification behavior and hallucination awareness, with different career-stage groups showing the greatest gaps for each outcome; they were broadly consistent across AI use frequency and publication volume. Formal research methodology training was the only factor significantly associated with improved disclosure behavior.

The high prevalence of AI use (60.3% reporting daily use) is consistent with international data documenting rapid integration of AI into medical research workflows [1,2]. Against this background, the finding that 40.2% of respondents had never encountered the concept of AI-generated fabricated references raises a practical concern: limited awareness of hallucination risk may reduce the likelihood of systematic verification among researchers who might otherwise verify if they understood the risk. This inference remains speculative given the cross-sectional and self-report nature of the data; actual verification behavior and its relationship to awareness would require prospective or observational methods to confirm.

The behavioral vignette data offer additional perspective. When presented with convincing-appearing AI-generated references, fewer than four in 10 respondents recognized the need for independent verification. When confronted with a discovered fabricated reference, 54.2% would remove only that citation and continue using surrounding AI output - a response that reflects an incomplete understanding that AI errors may affect any part of generated content, not only the specific citation identified. These responses are consistent with the broader self-reported verification patterns and together support the observation that high adoption coexists with underdeveloped critical appraisal habits in this sample, a pattern consistent with automation bias - the tendency to over-rely on automated tools as they become embedded in habitual workflows [14].

This risk may be particularly relevant for medical students. Across the sample, the most common uses of AI were literature search, generating or finding references, interpreting data, and writing and editing manuscripts, tasks in which fabricated references or fact-conflicting hallucinations could directly enter academic work. Students may lack the clinical or research experience needed to recognize implausible content, which may partly explain why medical students showed the lowest rate of independent verification of any professional group (Table 3); this explanation is speculative and was not tested in this study.

Publication volume, daily use frequency, and formal research training showed no statistically significant association with verification behavior in this sample. This suggests that verification habits in this population may be more strongly linked to career stage than to research experience, use intensity, or prior training alone - a pattern discussed further below in relation to the role-based findings.

The role-based findings warrant closer attention, as they were the most consistent statistically significant results in this dataset. Medical students showed the lowest rate of independent verification of any professional group, while residents showed the highest. This is consistent with this study's central concern: the earliest-career respondents, plausibly still forming their research habits, verified AI output the least. However, the parallel finding for hallucination awareness ran in a different direction - physicians and house officers, not medical students, showed the greatest gap in awareness of AI-generated fabricated references. This divergence suggests that the verification gap and the awareness gap, while related, may arise from different underlying mechanisms and are not simply two expressions of a single seniority effect. Further research using longitudinal or mixed-methods designs would be needed to clarify why verification and awareness follow different career-stage patterns in this population.

That nearly as many respondents reported no change in perspective (35.1%) as reported increased verification intent (46.4%) following survey completion tempers any interpretation of the survey itself as an effective intervention. While these results suggest that brief structured exposure to hallucination concepts may influence verification attitudes for a substantial subset of respondents, the absence of a longitudinal design and control condition precludes conclusions about the durability, causal nature, or generalizability of this effect.

The near-universal AI adoption observed in this sample (99.7%) contrasts sharply with the 40.3% adoption rate reported by Alturaiki et al. [10] in an international survey of 434 medical researchers across Saudi Arabia, Nigeria, Tunisia, and England conducted in 2023-2024. That study additionally found more senior roles associated with lower odds of use. This disparity likely reflects both the rapid acceleration of AI adoption between 2023-2024 and 2026, and this study's sample composition, which skewed toward medical students and early-career trainees rather than the broader career-stage distribution surveyed by Alturaiki et al. [10]. This contrast underscores that adoption rates in this literature are highly sensitive to sampling timeframe and population, complicating direct cross-study comparison.

The vignette finding that over half of respondents (54.2%) would continue using AI-generated content after discovering only one fabricated reference takes on additional significance in light of Chelli et al. [15], who found hallucination rates of 39.6% (GPT-3.5), 28.6% (GPT-4), and 91.4% (Bard) when these models were used to generate references for systematic reviews across 11 reviews and four fields. Given that fabrication rates of this magnitude are common even in GPT-4, the current study's finding that only 26.5% of respondents would verify all remaining AI-generated content after encountering a single fabricated reference suggests a meaningful mismatch between actual model reliability and researcher verification behavior.

The expanding role of AI extends beyond research and manuscript preparation into direct clinical practice. A 2026 international survey of 300 dermatologists across 13 countries found that 61.33% reported having used AI tools in their clinical work [16], suggesting that the verification concerns raised by this study may extend beyond academic manuscript preparation into direct patient care contexts, where the consequences of unverified AI-generated content could be more immediate.

The most robust finding in the inferential analysis was the strong association between formal research training and consistent AI disclosure (51.7% vs. 17.1%, p < 0.001). This suggests that structured exposure to research norms, including ethics, transparency, and publication standards, such as those articulated in current editorial guidance on AI disclosure [17], may support responsible AI use practices. Importantly, training was not significantly associated with verification behavior, pointing to a dissociation between normative awareness (knowing disclosure is required) and habitual practice (actually verifying content). This distinction may have practical implications for how AI literacy interventions are designed.

These findings may also be considered within a broader systemic context. One possible interpretation is that the observed discrepancy between adoption and responsible practice reflects, at least in part, the absence of institutional infrastructure; for example, the lack of a standardized AI literacy curriculum in Pakistani medical education, institutional verification protocols, or enforced disclosure norms. If this interpretation holds, structural interventions such as integrating AI literacy training into medical curricula and establishing institutional AI governance frameworks may be worth considering alongside efforts targeting individual behavior. However, this study did not directly measure institutional policies, curricula, or governance structures, nor did it compare the effectiveness of structural versus individual-level interventions; this remains a hypothesis for future research rather than a conclusion supported by the present data.

The apparent influence of survey participation on verification attitudes, with 46.4% reporting increased likelihood of verifying outputs after completing the survey, suggests that brief structured exposure to hallucination concepts may be sufficient to shift attitudes. This finding is preliminary and should not be overinterpreted; the absence of a control condition and longitudinal follow-up means the durability and causal nature of this effect cannot be established from these data.

This study has several limitations. The convenience sampling approach, conducted through WhatsApp, Instagram, Facebook, and LinkedIn networks, likely overrepresents digitally connected, research-engaged individuals - a bias directly evidenced by this sample's unusually high rate of prior research participation (97.0%), which is not representative of medical professionals generally. The cross-sectional design precludes causal inference and relies entirely on self-report, introducing potential recall and social desirability bias, particularly for sensitive items such as disclosure practices.

The questionnaire was not formally validated through psychometric testing; while it was reviewed by two co-investigators and refined iteratively during initial data collection, no independent, standardized validation process was conducted, and construct validity cannot be assured. In particular, no formal content validation, cognitive interviewing, or assessment of question comprehension was conducted prior to or during deployment; the two-co-investigator review and iterative refinement described in Methods do not substitute for these more rigorous validation steps, particularly for disclosure practice, one of this study’s principal outcomes, which was introduced only after data collection had already begun. The 71.0% concentration of respondents in Punjab province, with no representation from Balochistan or Gilgit-Baltistan, limits generalizability beyond this sample's specific geographic and professional composition. The ranking of Claude as the most commonly reported tool likely reflects investigator network characteristics and should not be generalized to national patterns.

No a priori sample size calculation was performed, and no corrections for multiple comparisons were applied given the exploratory nature of this study. Finally, this study measured self-reported perceptions and behaviors, not directly observed research conduct; actual rates of verification and fabricated reference use may differ from those reported here. Findings should be interpreted as hypothesis-generating rather than confirmatory. These findings should not be interpreted as representative of all physicians or researchers in Pakistan. Additionally, all associations reported are unadjusted bivariate analyses; no multivariable adjustment for potential confounding among demographic and behavioral variables was performed, and observed associations, including the role-based findings, may partly reflect the influence of correlated but unadjusted covariates such as training or publication history. The exclusion of the single “Other” role category from inferential analyses due to small subgroup size, described in the "Statistical analysis" section, is a related constraint on the role-based findings specifically.

## Conclusions

This cross-sectional survey suggests that high AI adoption coexists with underdeveloped verification habits, incomplete hallucination awareness, and inconsistent disclosure practices among medical trainees and professionals in Pakistan. Formal research methodology training was significantly associated with consistent AI disclosure, though not with verification behavior - a dissociation that may inform the design of future educational interventions. These findings suggest that AI literacy training, including explicit instruction on hallucination risk and verification practice, may be a worthwhile area for future research and potential intervention within Pakistani medical education curricula and institutional research governance frameworks; this study did not test the effectiveness of any such intervention. Replication using probability-based sampling across broader LMIC contexts is warranted.

## Appendices

The full survey instrument, including item wording and response options for all 24 items, is provided below.

**Table 5:** Full survey instrument used in the study (24 items across four domains). Items 22-24 (gender, purpose of AI use, and disclosure practice) were added after responses from the first 24 respondents were collected. Those initial participants were individually recontacted to complete the additional items before final data analysis.

Item	Domain	Survey question	Response options
1	Consent	I confirm that I am at least 18 years old, my participation is voluntary, and I understand that responses are anonymous.	I agree and wish to continue
2	Eligibility	Are you currently involved in medical education, clinical practice, research, or academic work in Pakistan?	Yes; No
3	Demographic	Current role	Medical student; House officer; Resident; Physician; Faculty; Other
4	Demographic	City	Free list of Pakistani cities; Other (please specify)
5	Demographic	Have you previously participated in research?	Yes; No
6	Demographic	Approximate number of publications	0; 1-2; 3-5; More than 5
7	Demographic	Have you received any formal training in research methodology?	Yes; No; Unsure
8	AI adoption	How frequently do you use AI tools for research or academic work?	Daily; Weekly; Monthly; Rarely; Never
9	AI adoption	Which AI tool do you use most often?	Claude; ChatGPT; Perplexity; Gemini; Other; I do not use AI
10	Verification	When using AI for research or academic work, which statement best reflects your usual approach?	Verify nearly all AI-generated content; Verify selected outputs or references only; Usually do not verify; Trust scientifically convincing outputs; Rarely or never use AI
11	Vignette 1	You use AI to generate a paragraph for a manuscript containing references that appear authentic. What would you assume?	Verify independently because AI may fabricate citations; Probably correct but important ones should be checked; Likely authentic if scientifically convincing; Never considered this possibility before
12	Vignette 2	Imagine you discover one fabricated reference in AI-generated content. What would be your most likely reaction?	Verify all remaining content; Remove only the incorrect reference and continue; Continue because occasional mistakes are expected; Become less willing to trust AI-generated content; Fabricated references are not a major concern
13	Hallucination awareness	Before today, which statement best described your understanding of AI-generated scientific errors?	Fully aware; Knew AI makes mistakes but not fabricated references; Never heard AI can fabricate scientific information; Rarely or never use AI
14	Verification	Have you ever personally encountered AI-generated scientific information that proved incorrect, fabricated, misleading, or unsupported after verification?	Yes, changed how I use AI; Yes, but no change; Suspected but never verified; No; Never checked
15	Verification	Which statement best reflects your current belief about your ability to safely use AI in research?	Usually identify errors without verification; Selective verification preferred; Cannot identify errors without independent verification; Never considered this; Rarely use AI
16	Verification	If AI increases efficiency but also the possibility of unnoticed scientific errors, which approach would you choose?	Prioritize reliability; Accept moderate gains with verification; Prioritize speed; Rely on peer review; Never considered this trade-off
17	Verification	Which AI-generated outputs would you consider acceptable without independent verification? (Select all that apply)	Grammar/readability; Summaries; Scientific explanations; Reference suggestions; Direct AI-generated references; Discussion sections; Verify everything; Never considered this
18	Verification	Which research tasks would you allow AI to perform with minimal or no independent verification? (Select all that apply)	Brainstorming; Language editing; Summaries; Selecting references; Data extraction; Interpretation; Conclusions; None; Never considered this
19	Attitudes	Which statement best reflects your view of AI-generated scientific content?	Reliable if convincing; Useful but should usually be verified; Never trust without verification; Personal experience is sufficient; Never considered this
20	Attitudes	Which statement best describes your current use of AI in research?	Improved efficiency and verification; More productive without changing critical appraisal; Faster but verify less; No change; Never reflected on this
21	Attitudes	After completing this survey, has your perspective on verifying AI-generated scientific content changed?	More likely to verify; No change; Already routinely verify; Unsure
22	Demographic	Gender	Female; Male
23	AI adoption	For which tasks do you primarily use AI in research or academic work? (Select all that apply)	Literature search; Generating/finding references; Data interpretation; Writing/editing manuscripts; Brainstorming; Structuring manuscripts; I do not use AI
24	Disclosure	When AI assists in preparing a manuscript or research document, do you disclose its use?	Yes, always; Sometimes; Never; I was not aware this is required; I do not use AI for manuscripts
